# Supplementary material for: Costs incurred by people with co-morbid tuberculosis and diabetes and their households in the Philippines
Source: PLoS One. 2024 Jan 25;19(1):e0297342. doi: 10.1371/journal.pone.0297342 (PMC10810501; doi:10.1371/journal.pone.0297342)
Supplement: S1 Text — (DOCX) [file pone.0297342.s001.docx]

In an over-arching cohort study (Effects of malnutrition and diabetes on treatment outcome and total patient costs in Filipino drug resistant and drug sensitive patients starting anti - TB treatment: A cohort study) study participants aged 18 or more (adults) who were initiating a new TB treatment regimen were recruited from participating NTP DOTs and iDOTS centres (i.e. those implementing the WHO shorter regimen to eligible DR-TB patients) within the National Capital Region (NCR), Negros Occidental and Cebu. Selected sites included:

**Manila, NCR (not used for our study sites):** San Lazaro Hospital**,** San Nicholas Health Centre

**Negros Occidental, Western Visayas (used for our study sites):** Valladolid Health Centre**,** Bago City Health Centre**,** Bacolod Health Centre**,** La Carlota Health Centre**,** Pablo O. Torres Memorial Hospital

**Cebu, Central Visayas (used for our study sites):** Compostela Health Centre**,** Carmen Health Centre**,** Consolacion Health Centre**,** Eversley Childs Sanitarium and General Hospital**,** Lapu-Lapu Health Centre**,** Vicente Sotto Hospital
