# Supplementary material for: Costs incurred by people with co-morbid tuberculosis and diabetes and their households in the Philippines
Source: PLoS One. 2024 Jan 25;19(1):e0297342. doi: 10.1371/journal.pone.0297342 (PMC10810501; doi:10.1371/journal.pone.0297342)
Supplement: S1 Table — (DOCX) [file pone.0297342.s002.docx]

|  | **Coefficients** | **Standard error** | **p-value** |
| --- | --- | --- | --- |
| **(constant)** | 32.4 | 36.7 | 0.377 |
| **Motorcycle/Tricycle** | -45.5 | 31.6 | 0.151 |
| **Car/Jeep/Van** | 427.2 | 86.7 | <0.001 |
| **Air conditioner** | 116.9 | 81.3 | 0.151 |
| **Stove with oven/Gas range** | 74.8 | 28.7 | 0.010 |
| **Refrigerator/Freezer** | 63.0 | 33.6 | 0.062 |
| **PC/tablet** | 171.7 | 52.5 | 0.001 |
| **Gold/jewellery** | 81.5 | 50.8 | 0.109 |
| **Number of rooms** | 29.2 | 12.6 | 0.021 |
