# Supplementary material for: Costs incurred by people with co-morbid tuberculosis and diabetes and their households in the Philippines
Source: PLoS One. 2024 Jan 25;19(1):e0297342. doi: 10.1371/journal.pone.0297342 (PMC10810501; doi:10.1371/journal.pone.0297342)
Supplement: S2 Table — (DOCX) [file pone.0297342.s003.docx]

|  | **TB people without diabetes** | | **TB people with diabetes** | | **Overall** | |
| --- | --- | --- | --- | --- | --- | --- |
|  | **%** | **(95% CI)** | **%** | **(95% CI)** | **%** | **(95% CI)** |
| **Social consequences** |  |  |  |  |  |  |
| Before TB diagnosis |  |  |  |  |  |  |
| Food insecurity | 3.6 | (2.2-6.0) | 1.7 | (0.2-11.5) | 3.4 | (2.0-5.5) |
| Divorce/separation | 0.3 | (0.04-1.8) | - | 0.0 | 0.2 | (0.03-1.6) |
| Job loss | 47.4 | (42.4-52.4) | 37.3 | (25.7-50.5) | 46.1 | (41.5-50.7) |
| Interrupted schooling | 0.5 | (0.1-2.1) | - | 0.0 | 0.4 | (0.1-1.8) |
| Social exclusion | 13.5 | (10.4-17.3) | 8.5 | (3.5-19.1) | 12.8 | (10.0-16.3) |
| End of intensive phase |  |  |  |  |  |  |
| Food insecurity | 8.0 | (5.7-11.2) | 5.1 | (1.6-14.9) | 7.6 | (5.5-10.5) |
| Divorce/separation | - | N/A | - | N/A | - | N/A |
| Job loss | 40.7 | (35.9-45.7) | 42.4 | (30.2-55.5) | 40.9 | (36.4-45.6) |
| Interrupted schooling | 0.8 | (0.2-2.4) | 1.7 | (0.2-11.5) | 0.9 | (0.3-2.4) |
| Social exclusion | 4.7 | (3.0-7.3) | 5.1 | (1.6-14.9) | 4.7 | (3.1-7.1) |
| Middle of continuation phase |  |  |  |  |  |  |
| Food insecurity | 6.2 | (4.2-9.1) | 3.4 | (0.8-12.9) | 5.8 | (4.0-8.5) |
| Divorce/separation | - | N/A | - | N/A | - | N/A |
| Job loss | 25.9 | (21.8-30.5) | 22.0 | (13.1-34.7) | 25.4 | (21.6-29.7) |
| Interrupted schooling | 0.3 | (0.04-1.8) | 1.7 | (0.2-11.5) | 0.4 | (0.1-1.8) |
| Social exclusion | 3.4 | (2.0-5.7) | 6.8 | (2.5-17.0) | 3.8 | (2.4-6.1) |
| End of continuation phase |  |  |  |  |  |  |
| Food insecurity | 4.4 | (2.7-7.0) | 3.4 | (0.8-12.9) | 4.3 | (2.7-6.6) |
| Divorce/separation | - | N/A | - | N/A | - | N/A |
| Job loss | 14.2 | (11.1-18.1) | 15.3 | (8.0-27.1) | 14.4 | (11.4-18.0) |
| Interrupted schooling | 0.3 | (0.04-1.8) | - | 0 | 0.2 | (0.03-1.6) |
| Social exclusion | 1.0 | (0.4-2.7) | 1.7 | (0.2-11.5) | 1.1 | (0.5-2.7) |
